# Hereditary ATTR Amyloidosis in Austria: Prevalence and Epidemiological Hot Spots

**DOI:** 10.3390/jcm9072234

**Published:** 2020-07-14

**Authors:** Michaela Auer-Grumbach, Rene Rettl, Klemens Ablasser, Hermine Agis, Christian Beetz, Franz Duca, Martin Gattermeier, Franz Glaser, Markus Hacker, Renate Kain, Birgit Kaufmann, Gabor G. Kovacs, Christian Lampl, Neira Ljevakovic, Jutta Nagele, Gerhard Pölzl, Stefan Quasthoff, Bernadette Raimann, Helmut Rauschka, Christian Reiter, Volha Skrahina, Othmar Schuhfried, Raute Sunder-Plassmann, Nicolas D. Verheyen, Julia Wanschitz, Thomas Weber, Reinhard Windhager, Raphael Wurm, Friedrich Zimprich, Wolfgang N. Löscher, Diana Bonderman

**Affiliations:** 1 Department of Orthopaedics and Trauma Surgery, Medical University of Vienna, 1090 Vienna, Austria; neiraljevakovic@hotmail.com (N.L.); reinhard.windhager@meduniwien.ac.at (R.W.); 2 Department of Cardiology, Medical University of Vienna, 1090 Vienna, Austria; rene.rettl@meduniwien.ac.at (R.R.); franz.duca@meduniwien.ac.at (F.D.); 3 Department of Cardiology, Medical University of Graz, 8036 Graz, Austria; klemens.ablasser@medunigraz.at (K.A.); nicolas.verheyen@medunigraz.at (N.D.V.); 4 Department of Hematology & Hemostaseology, Medical University of Vienna, 1090 Vienna, Austria; hermine.agis@meduniwien.ac.at; 5 Centogene AG, 18055 Rostock, Germany; Christian.Beetz@centogene.com (C.B.); volha.skrahina@centogene.com (V.S.); 6 Landesklinikum Waidhofen/Ybbs, 3340 Waidhofen an der Ybbs, Austria; martin.gattermeier@waidhofen-ybbs.lknoe.at; 7 Department of Internal Medicine, University of Krems, 3500 Krems, Austria; franz.glaser@lknoe.at (F.G.); Birgit.Kaufmann@krems.lknoe.at (B.K.); Bernadette.raimann@lknoe.at (B.R.); 8 Division of Nuclear Medicine, Medical University of Vienna, 1090 Vienna, Austria; markus.hacker@meduniwien.ac.at; 9 Department of Pathology, Medical University of Vienna, 1090 Vienna, Austria; renate.kain@meduniwien.ac.at; 10 Department of Laboratory Medicine and Pathobiology and Centre for Research in Neurodegenerative Disease, University of Toronto, Toronto, ON M5T 0S8, Canada; gabor.kovacs@uhnresearch.ca; 11 Institute of Neurology, Medical University of Vienna, 1090 Vienna, Austria; 12 Department of Neurology, Konventhospital der Barmherzigen Brüder Linz, 4021 Linz, Austria; christian.lampl@ordensklinikum.at; 13 Office for Internal Medicine, Spittal/Drau, 9800 Spittal an der Drau, Carinthia, Austria; jutta.nagele@gmail.com; 14 Department of Cardiology, Medical University of Innsbruck, 6020 Innsbruck, Austria; gerhard.poelzl@tirol-kliniken.at; 15 Department of Neurology, Medical University of Graz, 8036 Graz, Austria; stefan.quasthoff@medunigraz.at; 16 Department of Neurology, SMZ-Ost Hospital, 1220 Vienna, Austria; helmut.rauschka@wienkav.at; 17 Karl Landsteiner Institute for Neuroimmunological and Neurodegenerative Disorders/SMZ-Ost Hospital, 1220 Vienna, Austria; 18 Department of Cardiology, Kepler University Hospital, Medical Faculty, Johannes Kepler University, 4040 Linz, Austria; christian.reiter@kepleruniklinikum.at; 19 Department of Physical Medicine, Rehabilitation and Occupational Medicine, Medical University of Vienna, 1090 Vienna, Austria; othmar.schuhfried@meduniwien.ac.at; 20 Department of Laboratory Medicine, Medical University of Vienna, 1090 Vienna, Austria; raute.sunder@yahoo.at; 21 Department of Neurology, Medical University of Innsbruck, 6020 Innsbruck, Austria; julia.wanschitz@i-med.ac.at (J.W.); wolfgang.loescher@i-med.ac.at (W.N.L.); 22 Department of Internal Medicine II (Cardiology, Intensive Care Medicine), Klinikum Wels-Grieskirchen, 4600 Wels, Austria; thomas.weber@klinikum-wegr.at; 23 Department of Neurology, Medical University of Vienna, 1090 Vienna, Austria; raphael.wurm@meduniwien.ac.at (R.W.); friedrich.zimprich@meduniwien.ac.at (F.Z.)

**Keywords:** *TTR*, amyloidosis, prevalence, cardiomyopathy, polyneuropathy, transthyretin, Austria

## Abstract

Background: Hereditary transthyretin amyloidosis (hATTR) is an autosomal dominantly inherited disorder caused by an accumulation of amyloid fibrils in tissues due to mutations in the transthyretin (*TTR*) gene. The prevalence of hATTR is still unclear and likely underestimated in many countries. In order to apply new therapies in a targeted manner, early diagnosis and knowledge of phenotype-genotype correlations are mandatory. This study aimed to assess the prevalence and phenotypic spectrum of hATTR in Austria. Methods: Within the period of 2014–2019, patients with ATTR-associated cardiomyopathy and/or unexplained progressive polyneuropathies were screened for mutations in the *TTR* gene. Results: We identified 43 cases from 22 families carrying 10 different *TTR* missense mutations and confirmed two mutational hot spots at c.323A>G (p.His108Arg) and c.337G>C (p.Val113Leu). Two further patients with late onset ATTR carried *TTR* variants of unknown significance. The majority of patients initially presented with heart failure symptoms that were subsequently accompanied by progressive polyneuropathy in most cases. A total of 55% had a history of carpal tunnel syndrome before the onset of other organ manifestations. Conclusions: Our study underlined the relevance of hATTR in the pathogenesis of amyloid-driven cardiomyopathy and axonal polyneuropathy and indicated considerable genetic heterogeneity of this disease in the Austrian population. The estimated prevalence of hATTR in Austria based on this study is 1:200,000 but a potentially higher number of unknown cases must be taken into account. With respect to new therapeutic approaches, we strongly propose genetic testing of the *TTR* gene in an extended cohort of patients with unexplained heart failure and progressive polyneuropathy.

## 1. Introduction

Hereditary transthyretin amyloidosis (hATTR) is a rare, debilitating multisystem disorder caused by an extracellular deposition of transthyretin amyloid fibrils due to mutations in the transthyretin (*TTR*) gene. The most common manifestations affect the peripheral nerves and the heart, resulting in progressive amyloid polyneuropathy and hypertrophic amyloid cardiomyopathy. Involvement of other organs may also occur [1,2]. While mild to severe gastrointestinal problems are frequent, ocular and central nervous manifestations are rare. As an initial symptom, carpal tunnel syndrome (CTS) has been reported in up to 33% of patients with a mean period of 4–6 years before other organs become clinically involved [3]. More than 130 pathogenic mutations in the *TTR* gene associated with a high range of genotype–phenotype variability have been described [4]. A few mutations (mainly p.Val50Met, p.Glu109Gln and p.Val142Ile) are recurrent and occur endemically [5,6]. However, the global prevalence of hATTR is still unknown and might be underestimated. In fact, the THAOS registry collecting phenotypes and genotypes of ATTR in continental Western Europe neither covered the mutational landscape in Austria nor in Eastern Europe [7].

Moreover, wild-type TTR is intrinsically amyloidogenic and causes amyloid fibril formations in elderly individuals, resulting in senile systemic amyloidosis [3].

The significant progress in the treatment of ATTR by development of TTR tetramer stabilisers and gene silencing therapies suppressing variant and wild-type TTR synthesis now requires quick and early diagnosis of patients with both hATTR and wild-type ATTR and demands an improved understanding of phenotype-genotype correlations related to mutations [8,9,10].

In this study, we report clinical and genetic data of 22 Austrian families, carrying 10 different pathogenic or likely pathogenic variants in *TTR* and highlight the relevance of two possible *TTR* hotspot mutations in Austria.

## 2. Methods

### 2.1. Study Design and Population

Within the period of 2014–2019, patients with ATTR-associated and hypertrophic cardiomyopathy and/or progressive axonal peripheral neuropathies were screened for mutations in the *TTR* gene. Patients with peripheral neuropathies were either included when they had a positive family history or when all common causes had been excluded. All probands were examined by experienced cardiologists, neurologists and neurophysiologists at their primary care centres, which were located in different Austrian provinces. Visits were routinely scheduled every 6 months or more frequently when appropriate, as judged by the clinician. Additional at-risk family members were examined when available. The study protocol complies *TTR* with the Declaration of Helsinki and was approved by the ethics committee of the Medical University in Vienna (Ethics committee identification numbers: 796/2010 and 1738/2012). All patients gave written informed consent for participation in the study and were prospectively followed.

### 2.2. Diagnosis of Cardiac Amyloidosis

Diagnosis of ATTR was either made by endomyocardial biopsy (EMB) or non-invasively according to the algorithm by Gillmore et al. [11,12]. Cardiac magnetic resonance (CMR) was carried out in selected cases and was performed including gadolinium contrast application, T1 mapping and calculation of extracellular volume, as previously described [13,14]. The N-terminal prohormone of brain natriuretic peptide (NT-proBNP) plasma levels were analyzed in all patients. Severity of heart failure was scaled according to New York Heart Association (NYHA) guidelines. NYHA stage was documented at each visit.

### 2.3. Neurological Work-Up

Neurological examination included an assessment of motor, cerebellar and reflex function and a screening of all sensory nerve modalities (light touch, pinprick, vibratory, joint position sense and temperature sensation). Muscle strength was documented according to the Medical Research Council (MRC) scale (5/5 normal strength, 0/5 no contraction) in the majority of the patients. Motor and sensory nerve conduction studies (NCS) were performed according to standard procedures. The severity of polyneuropathy, when present, was classified according to international recommendations into stages 0–3 [15]. As the dataset was only documented to a limited extent in some medical records, the NIS + 7 score, which is the gold standard for documenting the course of the disease, could not be assessed for all patients [16]. Therefore, to describe degree and progression, further subdivision was made according to the modified polyneuropathy disability (PND) score, which was created based on data available for all patients (Stage 1: PND: I = sensory disturbances in extremities but preserved walking capacity; I = difficulties in walking but without the need for a walking stick; Stage 2: PND: IIIa = one stick or one crutch required for walking; IIIb = two sticks or two crutches required for walking; Stage 3: PND: IV = patient confined to a wheelchair or bed) [17].

Autonomic testing was only carried out in selected cases because in the patients included here, dysfunction of the autonomic nervous system was not a relevant feature except for gastrointestinal disturbances, including weight loss.

### 2.4. Genetic Testing of the TTR Gene

Genomic DNA was isolated from the patients’ peripheral blood using the QIAsymphony^®^ (Qiagen, Hilden, Germany) according to the manufacturer’s suggestions. Primers were used for the amplification and/or sequencing of exons 1–4 and the adjoining regions of the *TTR* gene with the aid of the Phusion Green Hot Start II High-Fidelity PCR Master Mix (Thermo Fisher Scientific, Atlanta, GA, USA) according to the manufacturer’s suggestions at an annealing temperature of 60 °C. Following the digest of residual PCR primers with Exo SAP-IT Express (Thermo Fisher), the sequencing reaction was performed with 2 µL digested PCR products using the BigDye Terminator Mix Version 3.1 (Thermo Fisher) according to the manufacturer’s suggestions. Sequencing was performed on an Applied Biosystems 3130xl Genetic Analyzer (ThermoFisher) according to the manufacturer’s suggestions. Sequences of the *TTR* gene (NM_000371.3; NG_009490.1) were analyzed using the Applied BioSystems SeqScape software v4.0 and the DNADynamo Sequence Analysis Software. Primer sequences are available upon request. However, it must be acknowledged that both the methodology and the allocation to genomic testing were not carried out uniformly, since patients were recruited in different institutions in Austria and testing was carried out in different facilities. In most cases, patients were only tested once.

## 3. Results

### 3.1. Genetic Spectrum and Distribution in Austria

**Figure 1:**
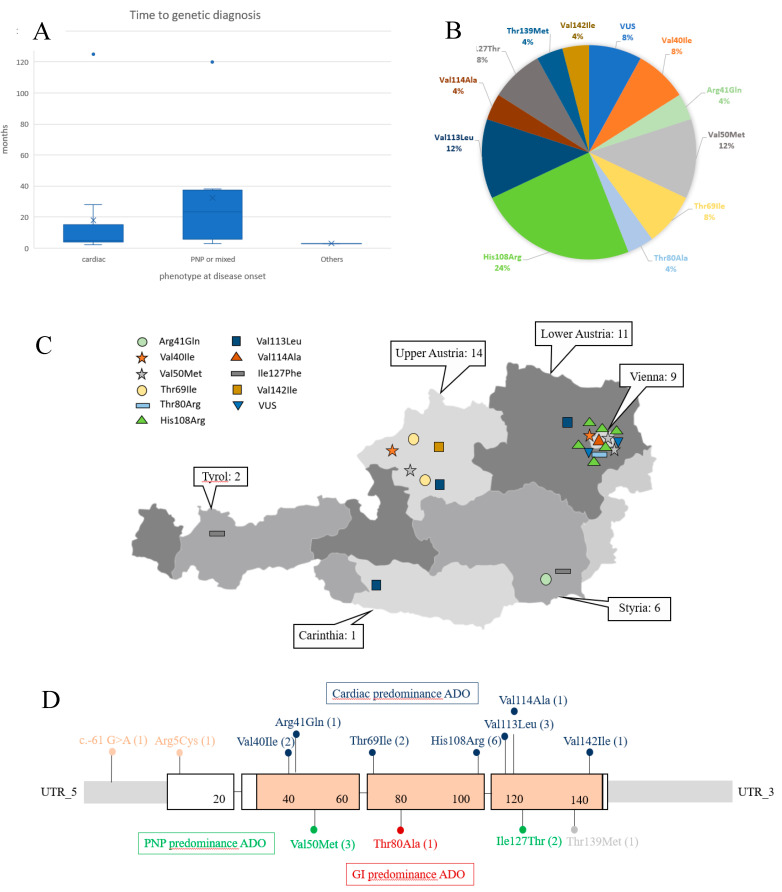
(**A**) Time to genetic diagnosis based on initial phenotype. (**B**) Genetic spectrum of *TTR* mutations observed in Austria. (**C**) Austrian map with geographic distribution of different genetic subtypes of hATTR. Numbers outside the map indicate the total of positively tested individuals, including pre-symptomatic mutation carriers. (**D**) Location of *TTR* mutations across the gene. Numbers in brackets indicate frequencies of the mutations observed in index patients. Predominant clinical consequences of the mutations are highlighted in different colors. PNP: polyneuropathy; GI: gastrointestinal; ADO: at disease onset.

In total, 10 different *TTR* missense mutations (p.Val40Ile (2×), p.Arg41Gln (1×), p.Val50Met (3×), p.Thr69Ile (2×), p.Thr80Ala (1×), p.His108Arg (6×), p.Val113Leu (3×), p.Val114Ala (1×), p.Ile127Phe (2×), p.Val142Ile (1×)) were detected in 43 individuals from 22 families (Figure 1B). Figure 1C shows the genetic spectrum and its geographic distribution across the Austrian map.

While most *TTR* mutations are spread across the country, there is a striking accumulation of the most common *TTR* mutation (p.His108Arg) in and around Vienna. Most patients except for two were native Austrians. One patient who carried the p.Thr80Arg variant originated from Iran, and another patient with the p.Val40Ile mutation was from Syria. Both patients had moved to and settled in Austria.

### 3.2. Baseline Clinical Characteristics of Index Patients with TTR Mutations

**Figure 2:**
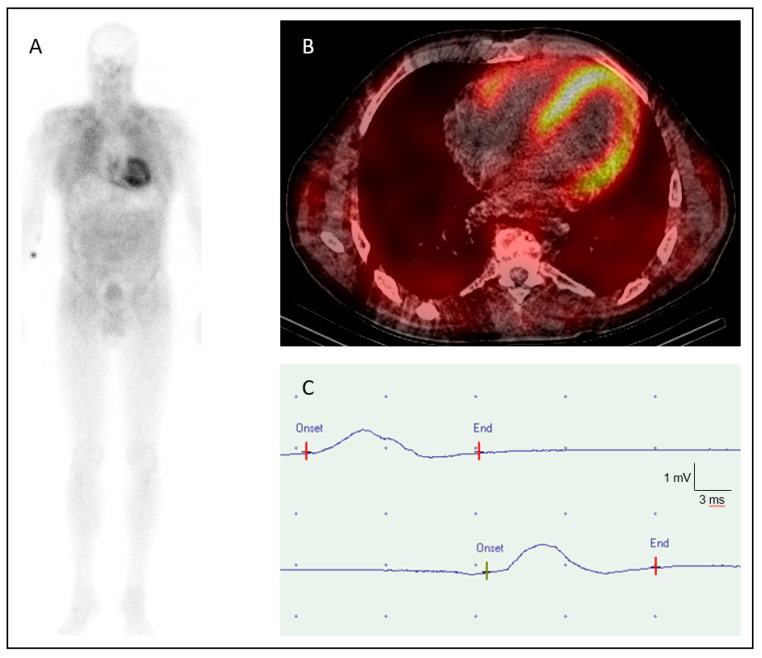
High-grade tracer uptake (Perugini grade 3) on (**A**) bone scintigraphy and (**B**) SPECT/CT (“low-dose” CT) with 99m-technetium-3,3-diphosphono-1,2-propanodicarboxylic acid (99mTc-DPD) in a patient with hATTR-associated cardiomyopathy (pVal40Ile) [18]. (**C**) Nerve conduction study of the right peroneal nerve of a patient with a p.His108Arg mutation (CMAP 0.6 mV, NCV: 30 m/sec).

The clinical and electrophysiological findings of the 22 index patients (12 males and 10 females) of each family are summarized in Table 1. In 12 cases (54.5%), dyspnea on exertion and limitation of physical capacity were the initial complaints that gave rise to a comprehensive internal and cardiological examination. TTE showed concentric a wall thickening compatible with cardiac ATTR amyloidosis, which was then confirmed by CMR, bone scintigraphy and/or EMB (Figure 2A,B). NT-proBNP levels were significantly elevated to 50 times of the normal value.

Another five patients (22.75%) were first brought to medical attention due to distal sensory disturbances and/or muscle weakness predominantly in the lower limbs. Subsequent neurological and electrophysiological assessment confirmed the presence of mild to moderate polyneuropathy ranging from stages 1/I to 1/II. Three patients (13.75%) presented with both cardiac and neurological features at the beginning of the disease and were thus classified as “mixed forms”. In one patient each (4.5%), investigation with regard to hATTR was initiated due to therapy-resistant diarrhea and vitreous opacity, respectively. Eleven patients (55%) had a history of uni- or bilateral carpal tunnel syndrome (CTS), which had been surgically treated 1–12 years before symptomatic onset of amyloidosis, except for one patient in whom CTS was diagnosed one and a half years after disease onset. Lumbar spinal stenosis was reported in only two patients but was not documented and questioned in all probands.

Variation in lag time between first examination and genetic diagnosis varied widely between 2–125 months and was usually shorter in patients with cardiac disease onset (Figure 1A).

All six patients carrying the p.His108Arg mutation initially presented with a predominant cardiac phenotype but developed considerable PNP with disease progression, and 5 of 6 index patients had CTS prior to symptomatic onset of amyloidosis. All three cases with the recurrent p.Val113Leu mutation presented with a predominant cardiac phenotype at disease onset.

Clinical, electrophysiological and radiological findings obtained in additional affected family members did not differ from those observed in index patients from the 22 families.

In two patients with late onset hypertrophic cardiomyopathy and mild polyneuropathy at 74 and 81 years due to ATTR amyloidosis, two rare *TTR* variants (c.-61G>A; p.Arg5Cys) of unknown significance (VUS) were identified.

### 3.3. Clinical Course within the Observation Period

While PNP was only detected in 45.5% at the first examination, 82% developed peripheral neuropathy with the progression of the disease as well as additional features, such as diarrhea and/or obstipation, weight loss (10 cases), and ocular and leptomeningeal involvement (1 case each). A persistent pure cardiological phenotype was observed in only three patients who carried the p.Val113 Leu (2 cases) and the p.Val142Ile (1 case) *TTR* mutations.

## 4. Discussion

This was the first study that determined the prevalence and the mutational spectrum of hATTR in the Austrian population counting 8,800,000 inhabitants. The detection of 43 individuals from 22 families carrying pathogenic or likely pathogenic *TTR* mutations indicated a similar frequency of ~1:200,000 in Austria when compared to estimates of 5.2 cases per 1 million in Europe apart from endemic regions in Portugal and Northern Sweden [19,20]. The repeated identification of Austrian hATTR families within the study period of 2014 to 2019 is strongly related to the increasing awareness of this disease due to new causative therapies and the facilitation of diagnoses [8,9,10]. Considering the potentially much higher number of undiagnosed patients due to on-going restricted genetic testing in some Austrian areas and to incomplete family studies, an even higher prevalence seems to be most likely. For example, in some regions of Austria, genetic testing is carried out on every patient with ATTR amyloidosis, while in other regions it is limited to patients with a positive family history. The continually underestimated frequency of hATTR in Austria is further supported by the rather broad spectrum of 10 different mutations in the *TTR* gene and the currently highly unequal distribution throughout Austria (Figure 1C).

Figure 1B,D provide an overview about frequencies and the location of all *TTR* mutations detected. While the most common p.Val50Met mutation was identified in only three families, there was a striking clustering of six families carrying the p.His108Arg variant in and around Vienna (Figure 1C). Given the fact that this mutation has been considered very rare and has so far mainly been observed in Italy and Sweden [7,21], it seems feasible that this particular Austrian hotspot mutation is due to a common founder. Expansion of the six families as well as genealogical and haplotype studies may serve to confirm or exclude this assumption. Further, the very rare p.Val113Leu mutation [7] requires special attention for a possible genetic hotspot, as it occurred in three independent index families in Austria.

Table 2 summarizes allele frequencies in the Genome Aggregation Database (gnomAD) and bioinformatic results for the identified *TTR* variants. As can be seen, all mutations in exons 2 to 4 are predicted to be pathogenic except for the p.Arg41Gln variant of unknown significance, which is characterised by a rather low Polyphen-2 score. Although no patients with hATTR carrying this mutation have been reported in the literature to our knowledge, the presence of cardiomyopathy in our patient, as well as its ultrarare occurrence in databases and its location within the gene beside clearly pathogenetic variants favor a possible disease-causing role of this variant. In addition, this variant has been listed as a pathogenic *TTR* mutation, affecting both the heart and the peripheral nerves in a *TTR* mutation database [22]. Still, this variant together with the two rare *TTR* variants (c.-61G>A; Arg5Cys) located outside the coding region and in exon 1 of the *TTR* gene, which is are associated with a very late onset mixed ATTR phenotype, require further confirmation. At present, we cannot exclude that these latter two variants of unknown significance occurred by chance in patients with wild-type ATTR.

Although the initial clinical phenotype was dominated by cardiomyopathy in more than 50% of our patients, the majority (82%) developed peripheral neuropathy soon after diagnosis and with disease progression, subsequently, other organs including eyes and brain became involved in selected cases.

This rather high proportion of predominant cardiac amyloidosis might be explained by a better awareness of hATTR among Austrian cardiologists, rather than among Austrian neurologists, which consequently results in more frequent genetic testing of patients with cardiomyopathy than of patients with unexplained axonal neuropathies. Further possible explanations also include the particular spectrum of *TTR* variants in Austria and more specific techniques, such as CMR, bone scintigraphy and/or EMB to detect cardiac amyloidosis.

There were no obvious specific features compared to previous clinical reports of hATTR. However, it must be highlighted that in our series of patients, CTS was a more common preceding disease event, as has been usually described [3].

In summary, this study contributed to understand and complete the genotypic variability of *TTR* mutations in Europe and reported two rare hot spot mutations in the *TTR* gene. It underscored the necessity to characterize mutations in Europe beyond countries considered in the THAOS registry and to facilitate documentation in disease-specific registries. In Austria, our results will have an impact on an improved awareness among healthcare professionals and also among patients and their families. This will assist in earlier and more accurate diagnosis which is indispensable for early applications of new therapies and a more effective management of hATTR patients.

## Figures and Tables

**Table 1:** Synopsis of clinical manifestations of pathogenic *TTR* variants.

Gender		Male = 12; Female = 10
Age at onset (median/range)		61.7 y/45–80 y (*n* = 22)
Current age (median/range)		66.9 y/56–82 y (*n* = 22)
Time until diagnosis (median/range)		21.8 m/2–125 m (*n* = 22)
Cardiac onset type		18.0 m/2–125 m (*n* = 12)
Other onset types		26.5 m/3–120 m (*n* = 10)
Initial presentation	Cardiac	54.50% (*n* = 12)
	PNP	22.75% (*n* = 5)
	Mixed	13.75% (*n* = 3)
	Gastrointestinal	4.50% (*n* = 1)
	Other	4.50% (*n* = 1)
NYHA Stage at first visit	1	9.00% (*n* = 2)
	2	64.00% (*n* = 14)
	3	9.00% (*n* = 2)
	No cardiac involvement	18.00% (*n* = 4)
PNP Stage/PND at first visit	1/I	22.75% (*n* = 5)
	1/II	22.75% (*n* = 5)
	2/IIIa, IIIb	0.00% (*n* = 0)
	3/IV	0.00% (*n* = 0)
	0 (No clinical signs of PNP)	54.50% (*n* = 12)
Current PNP Stage/PND	1/I	9.00% (*n* = 2)
	1/II	59.00% (*n* = 13)
	2/IIIa	4.50% (*n* = 1)
	2/IIIb	9.00% (*n* = 2)
	3/IV	0.00% (*n* = 0)
	0 (No clinical signs of PNP)	18.00% (*n* = 4)
Peroneal NCS	MNCV (mean/range)	39.2 m/s/29.5–49.5 m/s (*n* = 17) *
	CMAP (mean/range)	2.9 mV/0.4–9.1 mV (*n* = 17)
History of carpal tunnel syndrome	Unilateral	5.0 % (*n* = 1/20)
	Bilateral	50.0% (*n* = 10/20)
	Not present	45.0% (*n* = 9/20)
	Not known	9.0% (*n* = 2/22)
	Time before disease onset (y)	1–12 y

Abbreviations: CMAP = compound muscle action potential; MNCV = motor nerve conduction velocity; NCS = nerve conduction studies; PNP = polyneuropathy; m = months; y = years; Normal values: motor peroneal nerve: CMAP: 4.5–9.0 mV; NCV: 44.0–50.0 m/sec; * no response in one additional patient.

**Table 2:** Allele frequencies in gnomAD and bioinformatic results for the identified *TTR* variants.

SNP	c.DNA	Consequence	GnomAD ^1^				Polyphen2 Score ^2^	ClinVar	References
			Allele Count Total	Allele Frequency Total	Allele Count European (Non-Finish)	Allele Frequency Total (Non-Finish)			
rs770403822	c.-61G>A	5′UTR	10	0.0003184	4	0.0002592	n/a	Uncertain Significance	Illumina, SCV000408376.2
rs144792001	c.14G>A	p.Arg5His	36	0.0001273	22	0.0001704	0.274	Uncertain Significance	[7]
rs121918093	c.118G>A	p.Val40Ile	0	0	0	0	0.996	Pathogenic	^3^; [7]
rs879254269	c.122G>A	p.Arg41Gln	1	0.000003977	0	0	0.274	Uncertain Significance	^3^ PC
rs28933979	c.148G>A	p.Val50Met	26	0.0001034	19	0.0001670	1.000	Pathogenic	^3^; [7]
n/a	c.206C>T	p.Thr69Ile	n/a	n/a	n/a	n/a	1.000	n/a	^3^; [7]
rs121918070	c.238A>G	p.Thr80Ala	1	0.000003977	1	0.000008792	0.860	Pathogenic	^3^; [7]
n/a	c.323A>G	p.His108Arg	n/a	n/a	n/a	n/a	1.000	n/a	^3^; [21]
n/a	c.337G>C	p.Val113Leu	n/a	n/a	n/a	n/a	0.999	n/a	[7]
n/a	c.341T>C	p.Val114Ala	n/a	n/a	n/a	n/a	0.999	n/a	^3^
n/a	c.379A>T	p.Ile127Phe	n/a	n/a	n/a	n/a	0.996	n/a	^3^; [7]
rs28933981	c.416C>T	p.Thr139Met *	417	0.001474	351	0.002718	1.000	Likely benign	^3^
rs76992529	c.424G>A	p.Val142Ile	435	1.54E-03	4	0.00003098	1.000	Pathogenic	^3^; [7]

VUS: variant of unknown significance; n/a: no data. ^1^ GnomAD: frequency of *TTR* variants in Genome Aggregation Database (gnomAD); ^2^ Polyphen-2 scores* near 1 most strongly predict a “damaging” effect of an amino substitution, ^3^ referred in http://www.amyloidosismutations.com/ [22]; PC = personal communication. * In this study, this variant has been identified in one patient with unexplained axonal peripheral neuropathy. * Polyphen-2 is a tool that predicts a possible impact of an amino acid substitution on the structure and function of a human protein using straightforward physical and comparative considerations.
